# Mps1 (Monopolar Spindle 1) Protein Inhibition Affects Cellular Growth and Pro-Embryogenic Masses Morphology in Embryogenic Cultures of *Araucaria angustifolia* (Araucariaceae)

**DOI:** 10.1371/journal.pone.0153528

**Published:** 2016-04-11

**Authors:** Jackellinne C. Douétts-Peres, Marco Antônio L. Cruz, Ricardo S. Reis, Angelo S. Heringer, Eduardo A. G. de Oliveira, Paula M. Elbl, Eny I. S. Floh, Vanildo Silveira, Claudete Santa-Catarina

**Affiliations:** 1 Laboratório de Biologia Celular e Tecidual, Centro de Biociências e Biotecnologia (CBB), Universidade Estadual do Norte Fluminense Darcy Ribeiro (UENF), Campos dos Goytacazes, Rio de Janeiro, Brazil; 2 Laboratório de Biotecnologia Vegetal, Núcleo em Ecologia e Desenvolvimento Sócio-ambiental de Macaé, Universidade Federal do Rio de Janeiro, Macaé, Rio de Janeiro, Brazil; 3 Laboratório de Biotecnologia, CBB, UENF, Campos dos Goytacazes, Rio de Janeiro, Brazil; 4 Unidade de Biologia Integrativa, Setor de Proteômica, UENF, Campos dos Goytacazes, Rio de Janeiro, Brazil; 5 Laboratório de Biologia Celular de Plantas, Departamento de Botânica, Instituto de Biociências, Universidade de São Paulo, São Paulo, São Paulo, Brazil; Texas A&M University, UNITED STATES

## Abstract

Somatic embryogenesis has been shown to be an efficient tool for studying processes based on cell growth and development. The fine regulation of the cell cycle is essential for proper embryo formation during the process of somatic embryogenesis. The aims of the present work were to identify and perform a structural and functional characterization of Mps1 and to analyze the effects of the inhibition of this protein on cellular growth and pro-embryogenic mass (PEM) morphology in embryogenic cultures of *A*. *angustifolia*. A single-copy Mps1 gene named *AaMps1* was retrieved from the *A*. *angustifolia* transcriptome database, and through a mass spectrometry approach, AaMps1 was identified and quantified in embryogenic cultures. The Mps1 inhibitor SP600125 (10 μM) inhibited cellular growth and changed PEMs, and these effects were accompanied by a reduction in AaMps1 protein levels in embryogenic cultures. Our work has identified the Mps1 protein in a gymnosperm species for the first time, and we have shown that inhibiting Mps1 affects cellular growth and PEM differentiation during *A*. *angustifolia* somatic embryogenesis. These data will be useful for better understanding cell cycle control during somatic embryogenesis in plants.

## Introduction

The transition from a somatic cell into a somatic embryo, during somatic embryogenesis, is a complex event, consisting of the following crucial steps: induction, cell dedifferentiation, and competence acquisition; multiplication, with intense cell division; maturation, which determines fate; and the germination of somatic embryos [1].

During somatic embryo formation, the correct performance of the cell cycle is crucial, and adequate levels of certain signaling molecules, such as polyamines, carbohydrates, and nitric oxide (NO), are required [2–4]. The maturation induction of somatic embryogenic cultures with maturation promoters, such as abscisic acid (ABA), or with osmotic agents, such as polyethylene glycol (PEG) and maltose, induce cell growth inhibition, preventing division and promoting the differentiation of cell cultures [5–8]. However, other compounds, such as auxins, NO, and putrescine, promote cell division, thereby increasing growth and inhibiting cell differentiation into somatic embryos [4,6,7]. Embryogenic suspension culture systems have been developed for *Araucaria angustifolia*, and they have been shown to be efficient systems for studying the effects of signaling molecules in gymnosperms [4,9,10]. Cell cycle regulation can be used as a tool for the elucidation of metabolism-related events, and it involves signaling compounds that are important for various processes in plant development [11], including somatic embryogenesis [12].

Cell division in eukaryotes is controlled by a complex mechanism that involves cyclin-dependent kinases (CDKs) as key regulators [13,14]. One of these kinases is Mps1 (monopolar spindle 1), which has been described in humans and is characterized as a cell cycle regulator that is evolutionarily conserved in eukaryotes [15]. Mps1 is a dual-specificity protein kinase that plays a critical role in monitoring the accuracy of chromosome segregation at the mitotic checkpoint, and it is an important component of the spindle assembly checkpoint (SAC) [16]. Among chemical inhibitors, SP600125 acts on Jun N-terminal kinase (JNK) proteins in humans [17] and has been valuable in validating the cellular functions of Mps1. In plants, a protein was found that was highly similar to human Mps1 in terms of structural characteristics, such as its catalytic site, and it was conserved relative to the Mps1 protein found in *A*. *thaliana* [18]. The inhibitor SP600125 blocks the G2-M transition in *Arabidopsis* by specifically inhibiting the activity of AtMps1 [18]. However, the role of Mps1 in gymnosperm species, such as *A*. *angustifolia*, has not yet been shown.

The aims of the present work were to identify and perform a structural and functional characterization of Mps1 and to analyze the effects of the inhibition of this protein on cellular growth and pro-embryogenic mass (PEM) morphology in embryogenic cultures of *A*. *angustifolia*.

## Materials and Methods

### Plant Material

Embryogenic suspension cultures of *A*. *angustifolia* were induced according to the methodology established by Steiner et al. [19] and then used for these experiments. Embryogenic cell suspension cultures are formed by PEMs made of embryogenic cells (which are rounded, with a dense cytoplasm) and suspensor cells (which are highly vacuolated and elongated) [6,20].

### Mps1 Sequence Identification and Structural Analyses

To identify a putative Mps1 from *A*. *angustifolia*, we performed a tBLASTn search [21] by using the Mps1 protein sequence of *A*. *thaliana* (AT1G77720) as a query against the *A*. *angustifolia* transcriptome database [22,23], with the following parameters: E-value > E^-10^ and a minimum coverage threshold of 30% (query and hit). The complete sequence is available at GenBank under accession number KU600448. Other sequences that were homologous to their *A*. *thaliana* counterpart were identified by searching the Phytozome 10.2 database (http://www.phytozome.net/), NCBI (http://www.ncbi.nlm.nih.gov/), TAIR (https://www.arabidopsis.org), and SustainPineDB (http://www.scbi.uma.es/sustainpinedb) using BLAST. All the sequences obtained here and the putative *AaMps1* were aligned with MEGA software, version 6.0 [24] using MUSCLE/CLUSTALW with default parameters. The alignment was analyzed using the Neighbor-Joining method, and the distances were calculated according to the best model identified by the program. The model parameter and tree estimates were performed with PhyML [25], and the tree topology was evaluated with 1500 bootstrap replications. Detailed information on all the sequences used for analysis is available in S1 Table.

A template identification using the Mps1 sequence from *A*. *angustifolia* was performed using the template identification tool from SWISS-MODEL [26–28] to find the most accurate templates (by considering the sequence identity, coverage, and crystal resolution). Additionally, we performed a motif search analysis with the aid of the Eukaryotic Linear Motif (ELM) server [29] to find interaction sites with other cell cycle regulation elements.

Molecular modeling was performed using MODELLER v9.14 [30,31] with the following structures as templates: 2ZMD [32], 3DBQ [33], 3HMN [34], and 3VQU (http://dx.doi.org/10.2210/pdb3vqu/pdb). All four crystals are representations of the human Mps1 protein. Molecular docking experiments with the *A*. *angustifolia* Mps1 3D model were performed with Autodock v4.6.2 [35]. Experimental conditions were set using the oxygen atom (position 838) from the GLU-790 residue inside a 45x45x45 (XYZ dimensions) grid box centered at approximately 0.9460/-32.2960/-9.4240 (x/y/z coordinates). The molecular docking and modeling solutions were visualized and registered with PyMOL v1.3 (Schrödinger, LCC), using the Autodock plugin [36].

Linear protein interaction motifs were detected with the ELM Database (http://elm.eu.org/) [29]. The Mps1 proteins analyzed here were from the species *A*. *angustifolia* (AaMps1), *Amborella trichopoda* (AbMps1 –gi | 586646077), *Eucalyptus grandis* (EgMps1 –gi | 702379945), *Carica papaya* (CpMps1 -| evm.TU.supercontig_36.11), and *Medicago truncatula* (MtMps1 gi | 357461629). Phosphorylation sites were predicted with PlantPhos, a tool that was developed to predict phosphorylation sites in plant proteins [37].

### Suspension Culture Conditions

To obtain cell suspensions, embryogenic cultures were multiplied and maintained in the basic liquid culture medium MSG [38] supplemented with 30 g l^-1^ sucrose, 1.4 g l^-1^ L-glutamine (Sigma-Aldrich, St. Louis, USA), and 0.1 g l^-1^ myo-inositol (Merck KGaA, Darmstadt, Germany), and the pH of the culture medium was adjusted to 5.7 before autoclaving at 121°C for 20 min, 1.5 atm. The embryogenic cell suspension cultures were subcultured every 15 days by adding 10 ml of the old suspension culture to 60 ml of fresh liquid medium. Embryogenic cell suspension cultures were kept on an orbital shaker (Cientec, Minas Gerais, Brazil) at 100 rpm in the dark, at 25 ± 2°C.

To analyze the effect of Mps1 inhibition on cellular growth and the PEM morphology, embryogenic cell suspension cultures were grown in basic MSG culture medium supplemented with 30 g l^-1^ sucrose, 1.4 g l^-1^ L-glutamine, and 0.1 g l^-1^ myo-inositol, and with or without Mps1 inhibitor SP600125 (Sigma-Aldrich). The Mps1 inhibitor was filter-sterilized through a 0.2-μm PVDF membrane (Millipore, São Paulo, Brazil) before being added to the culture medium. After the inoculation of embryogenic cell culture with 15-day-old cell suspensions, the flasks were maintained on an orbital shaker at 100 rpm in the dark, at 25 ± 2°C.

### Effects of Mps1 Inhibition on Cellular Growth

The cellular growth in suspension cultures was measured using settled cell volume (SCV) according to Osti et al. [4] to establish the growth curve for different concentrations (0, 1 and 10 μM) of Mps1 inhibitor. The SCV was determined by cell sedimentation in the side arm of the adapted flasks and was evaluated every three days until day 30 of the culture. Each treatment was performed in triplicate. From the resulting growth curve, the initial time, lag phase, early exponential phase, exponential phase, and stationary phase were established as days 0, 6, 15, 21, and 27, respectively, of the incubation.

To analyze cellular growth based on increases in fresh matter (FM) and dry matter (DM), 60 mg aliquots of 15-day-old embryogenic suspension cultures were inoculated into 12-well tissue culture plates (TPP^®^) containing 2 ml/well of basic MSG culture medium without (control) or with (10 μM) Mps1 inhibitor. The application of Mps1 inhibitor (10 μM) inhibited the cellular growth according to SCV analyses. Six samples (corresponding to six wells) from each treatment were obtained to measure the FM before (0) and after 6, 15, 21, and 27 days of incubation. The DM was obtained by drying the FM samples at 70°C for 48 h.

### Effects of Mps1 Inhibition on PEM Morphology

The analyses of PEM morphology were performed by measuring the area and size of embryogenic cells and suspensor cells. For both analyses, samples were collected before (0) and after 6, 15, 21, and 27 days of incubation without (control) or with (10 μM) Mps1 inhibitor, which showed cellular growth inhibition in the SCV analyses. Samples were collected and prepared on slides, followed by examination under an Axioplan light microscope (Carl Zeiss, Jena, Germany) equipped with an AxioCam MRC5 digital camera (Carl Zeiss). After the images were obtained, area and size were measured using AxioVision LE software, version 4.8 (Carl Zeiss).

The area measurements were performed from PEMs, from the group of embryogenic-type cells that form the embryonal head, and from the suspensor-type cells. For these analyses, for each treatment and each incubation time, three slides were prepared, and at least ten images of PEMs were obtained.

For the cell size analyses, the PEMs were treated with cellulase (Fluka Analytical, Buchs, Switzerland) 0.1% for 3 h to dissociate the embryogenic and suspensor cells of PEM. As embryogenic-type cells are isodiametric, the size was measured based on the diameter, and as suspensor-type cells are elliptic and elongated, the size was measured using the length and width (at the middle of the cell). For these analyses, for each treatment and each incubation time, three slides were prepared, and fifty images from each cell type (embryogenic or suspensor) were obtained.

### Identification and Quantification of the AaMps1 Protein

The AaMps1 protein was identified and quantified using embryogenic suspension cultures before (time 0) and after 15 days of incubation (the period of cellular growth) without (control) and with Mps1 inhibitor (10 μM), which inhibited cellular growth. This analysis was performed to confirm the presence of this protein in the embryogenic suspension cultures and to observe the effect of the inhibitor on the protein concentration in the two treatments.

Protein extractions were performed according to Balbuena et al. [39] with some modifications. Samples containing 300 mg FM were ground in liquid nitrogen and transferred into clear 2 ml microtubes containing 1.0 ml of extraction buffer made of 7 M urea (GE Healthcare, Freiburg, Germany), 2 M thiourea (GE Healthcare), 1% dithiothreitol (DTT; GE Healthcare), 2% Triton X-100 (GE Healthcare), 0.5% pharmalyte (GE Healthcare), 1 mM phenylmethanesulfonyl fluoride (PMSF; Sigma-Aldrich), and 5 μM pepstatin (Sigma-Aldrich). All extracts were vortexed for 2 min and kept in the extraction buffer on ice for 30 min, followed by centrifugation at 12,000 x *g* for 10 min at 4°C. The supernatants were transferred to clear microtubes; then, the proteins were precipitated in ice for 30 min in 10% trichloroacetic acid (TCA; Sigma-Aldrich) and were washed three times with cold acetone (Merck). Finally, the proteins were re-suspended and concentrated in 1 ml of the same extraction buffer. The protein concentration was estimated using a 2-D Quant Kit (GE Healthcare). Sample preparation and HDMS^E^ (data-independent acquisition, with ion mobility) mass spectrometry analyses were performed according to Reis et al. [40].

MS data processing and database searching were performed using Progenesis QI for Proteomics Software V. 2.0 (Nonlinear Dynamics, Newcastle, UK). The analysis used the following parameters: 1 missed cleavage; minimum fragment ion per peptide equal to 1; minimum fragment ion per protein equal to 3; minimum peptide per protein equal to 1; variable modifications by carbamidomethyl (C), acetyl N-terminal, and oxidation (M); a default false discovery rate (FDR) value with a 4% maximum; a score greater than 5; and a maximum of 10 ppm for mass errors. This program compares the AtMps1 (*A*. *thaliana*) sequence—gi | 28416703 and the AaMps1 (*A*. *angustifolia*) predicted protein sequence obtained by BLAST with the *A*. *angustifolia* transcriptome database [22,23] for protein identification.

### Data Analysis

The data presented here were statistically analyzed using analysis of variance (ANOVA) (*P<* 0.01) followed by Tukey's test using R software (Foundation for Statistical Computing, version 3.0.3, 2014, Vienna, Austria). Nucleotide sequence data from this article can be found in the GenBank under accession number KU600448.

## Results

### Mps1 Sequence Identification and Structural Analyses

**Fig 1:**
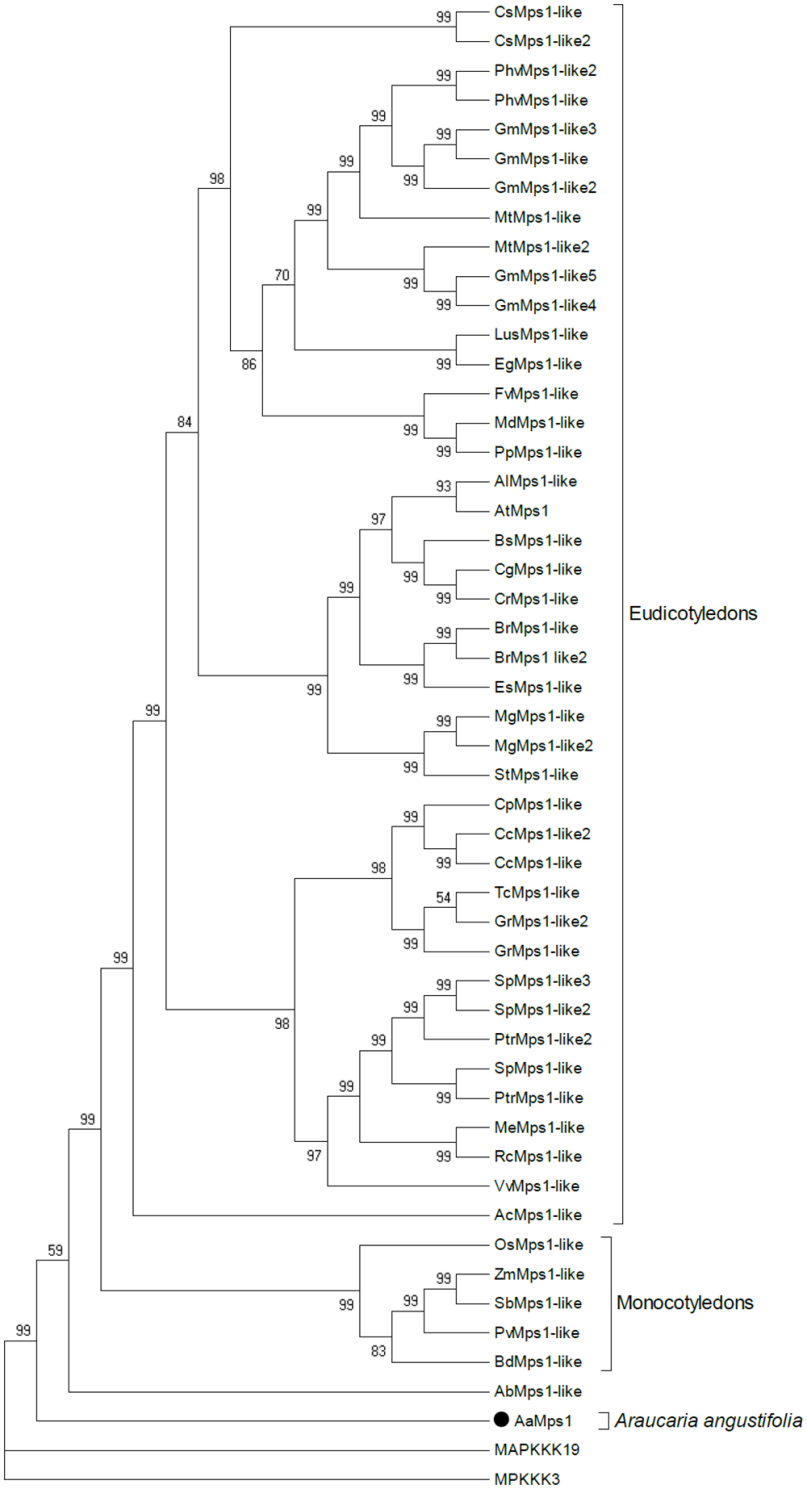
Phenogram of Mps1. Sequence data details are listed in S1 Table. The topology of the tree was consistent with the phylogenetic distribution of the species. Mps1 is encoded by a single-copy gene in monocotyledons and *Araucaria angustifolia*. Paralogs were found in some species inside the Eudicotyledons clade, indicating species-specific duplications. The bootstrap values are shown on the branches. The tree was rooted with MAPKs of *Arabidopsis thaliana* as the outgroup. *Amborella trichopoda* (Ab), *Aquilegia coerulea* (Ac), *Arabidopsis lyrata* (Al), *Arabidopsis thaliana* (At), *Araucaria angustifolia* (Aa), *Boechera stricta* (Bs), *Brachypodium distachyon* (Bd), *Brassica rapa* (Br), *Capsella grandiflora* (Cg), *Capsella rubella* (Cr), *Carica papaya* (Cp), *Citrus clementina* (Cc), *Cucumis sativus* (Cs), *Eucalyptus grandis* (Eg), *Eutrema salsugineum* (Es), *Fragaria vesca* (Fv), *Glycine max* (Gm), *Gossypium raimondii* (Gr), *Linum usitatissimum* (Lu), *Malus domestica* (Md), *Manihot esculenta* (Me), *Medicago trunculata* (Mt), *Mimulus guttatus* (Mg), *Oryza sativa* (Os), *Panicum virgatum* (Pv), *Phaseolus vulgaris* (Phv), *Pinus pinaster* (Ppi), *Populus trichocarpa* (Pt), *Prunus persica* (Pp), *Ricinus communis* (Rc), *Salix purpurea* (Sp), *Solanum tuberosum* (St), *Sorghum bicolor* (Sb), *Theobroma cacao* (Tc), *Vitis vinifera* (Vv), and *Zea mays* (Zm).

Using AtMps1 as a query, we identified a single-copy gene in *A*. *angustifolia*, and its protein was designated AaMps1 (Fig 1 and S1 Table). This sequence presented higher homology with AbMps1 protein in *Amborella trichopoda*, EgMps1 in *Eucalyptus grandis*, CpMps1 in *Carica papaya*, and MtMps1 in *Medicago truncatula* (S1 Fig).

**Fig 2:**
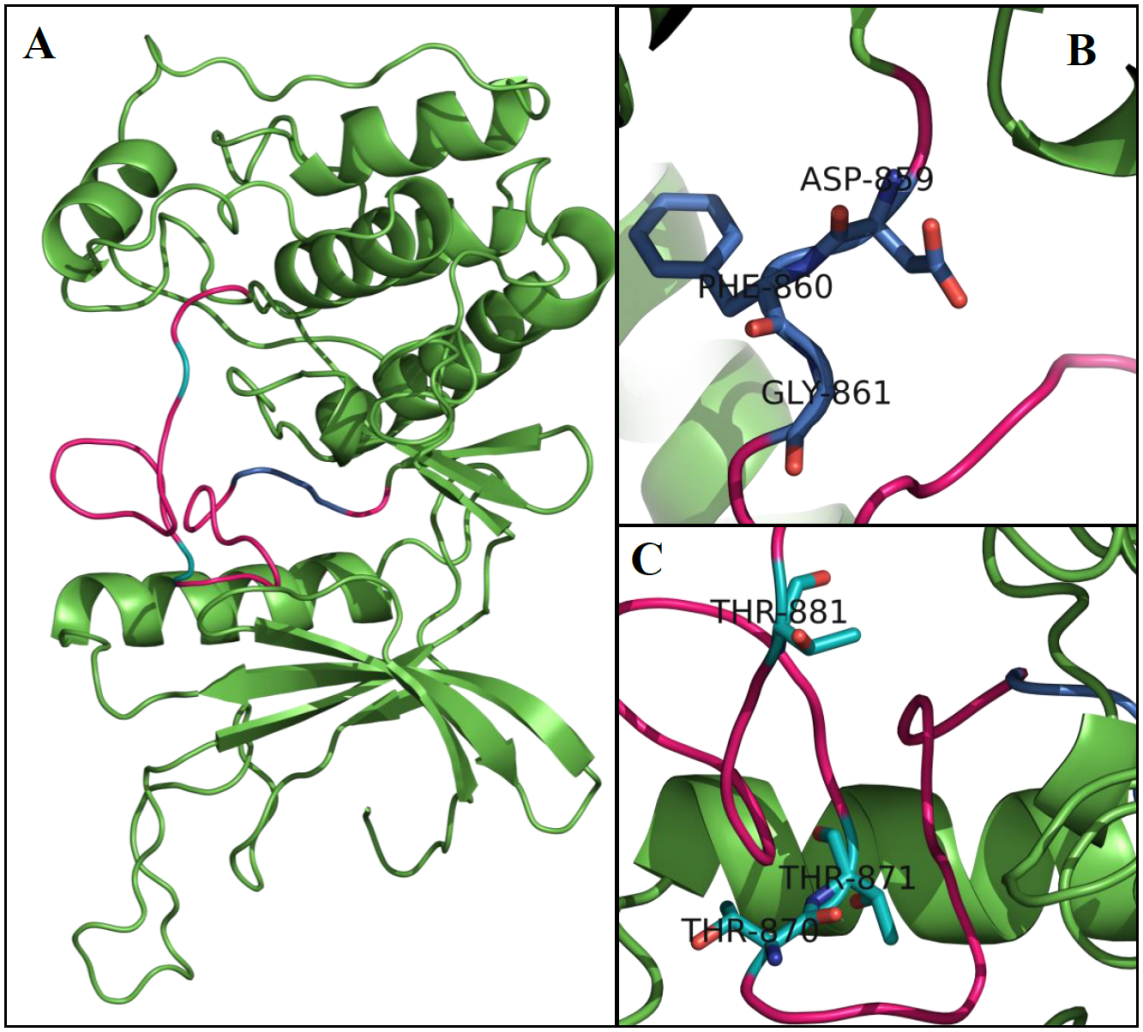
A 3D model of the AaMps1 kinase domain. (A) An overview of the kinase domain. Rose: activation loop; Blue: DFG motif; and Cyan: threonines. (B) A detailed view of the DFG motif. (C) A detailed view of the threonine residues (T870, T871, and T881) that are related to autophosphorylation.

A kinase domain with 293 amino acid residues could be identified, with approximately 91% of these amino acids being common between the different species, thus showing that this kinase domain is conserved among the analyzed species (S1 Fig). Tridimensional modeling of the AaMps1 kinase domain (Fig 2A) presents two subdomains that are connected by a flexible loop. The larger subdomain is composed of five α-helices and four β-sheets, and the smaller subdomain contains one α-helix and five β-sheets (Fig 2A). The alignment of the AaMps1 kinase domain reveals a structure that is similar to that of hMps1 (S2 Fig) and AtMps1 (S3 Fig). A tridimensional analysis of the AaMps1 kinase domain also showed that an Asp-Phe-Gly (DFG) motif (Fig 2B) and a threonine triad (T870, T871 and T881) related to autophosphorylation (Fig 2C) were highly conserved in other analyzed plant species (S1 Fig).

The phosphorylation sites in the kinase domain of the Mps1 protein were predicted (Table 1) using the AtMps1 sequence in PlantPhos, leading to the identification of 18 sites in AaMps1 that are analogous to the phosphorylation sites observed in AtMps1. In comparison with the AaMps1 sequence, 16 phosphorylation sites were predicted in EgMps1, 18 in CpMps1, and 17 in MtMps1.

**Table 1:** Phosphorylation sites of the kinase domain.

Residue Position	Residue Substrate	*A*. *angustifolia*	*A*. *thaliana*	*E*. *grandis*	*C*. *papaya*	*M*. *truncatula*
691	Y	X	X	X	X	X
699	S	X	X	-	X	X
702	S	X	X	X	X	X
703	S	X	X	X	X	X
710	S	X	X	X	X	X
711	S	X	X	X	X	X
714	T/S	X	X	X	X	-
716	Y	X	X	X	X	X
728	Y	X	X	X	X	X
732	Y	X	X	X	X	-
756	Y	X	X	-	X	-
786	Y	X	X	X	-	X
870	T	-	-	-	-	X
871	T	X	X	X	X	X
881	T	X	X	X	X	X
884	Y	X	X	X	X	X
922	Y	X	X	X	X	X
941	T	X	-	X	X	X
949	Y	X	X	-	X	X
953	S	X	X	X	X	X

Phosphorylation sites of the kinase domain in *A*. *angustifolia* AaMps1 analyzed by PlantPhos and compared with *A*. *thaliana* (AtMps1), *E*. *grandis* (EgMps1), *C*. *papaya* (CpMps1), and *M*. *truncatula* (MtMps1). Arrows indicate conserved threonine triads in the species. Y = tyrosine; S = serine; T = threonine; X = presence; and − = absence.

**Fig 3:**
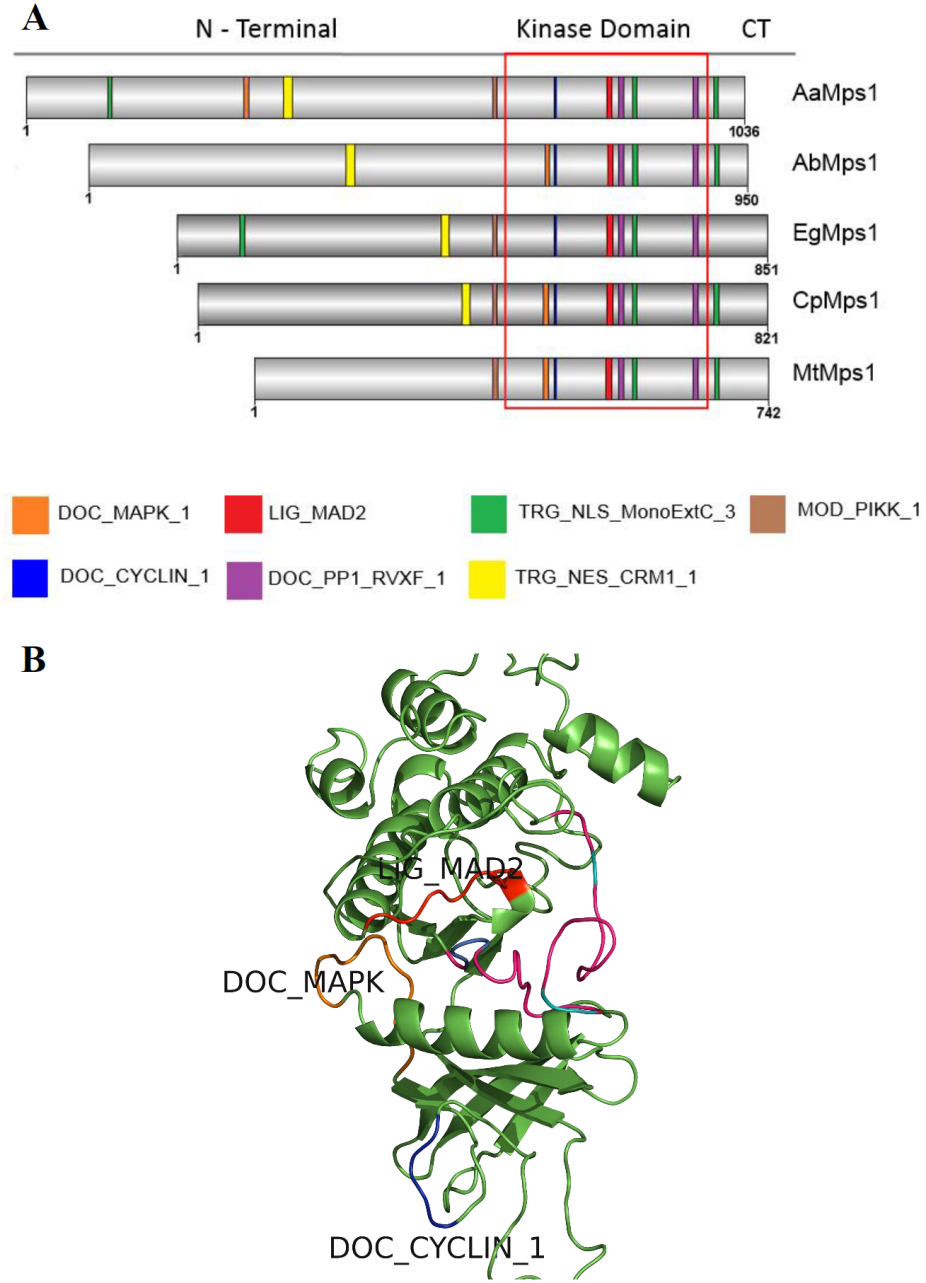
Mps1 motifs related to the cell cycle in plants. (A) Linear motifs of several Mps1 orthologs that were observed in *A*. *angustifolia* (AaMps1), *A*. *thicopoda* (AbMps1), *E*. *grandis* (EgMps1), *C*. *papaya* (CpMps1), and *M*. *truncatula* (MtMps1). (B) The 3D model of relevant interaction motifs DOC_CYCLIN_1, DOC_MAPK and LIG_MAD2 in AaMps1.

The AaMps1 sequence revealed 1036 residues, and the proteins AbMps1, EgMps1, CpMps1 and MtMps1 contained 950, 851, 821 and 742 residues, respectively (Fig 3A). The linear protein interaction motifs of Mps1 were analyzed with ELM prediction tool motifs to compare AaMps1 with Mps1 proteins from other species. AaMps1 had the characteristic motifs of the Mps1 protein kinase (Fig 3A), which were observed in *A*. *trichopoda*, *E*. *grandis*, *C*. *papaya*, and *M*. *truncatula*. The motifs that were found to be conserved in these species include the Mitotic arrest-deficient 2 (MAD2) binding motif LIG_MAD2, the Cyclin recognition site DOC_CYCLIN_1, the MAPK docking motif DOC_MAPK_1, the Nuclear Export Signal TRG_NES_CRM1_1, the Nuclear Localization Signal TRG_NLS_MonoExtC_3, the Protein phosphatase-1 (PP1) regulation site DOC_PP1_RVXF_1, a motif phosphorylated by phosphoinositide-3-OH-kinase (PIKK) family members, MOD_PIKK_1, and a motif for the DFG structural conformation. These motifs were present in at least four species among those analyzed here (AaMps1, AbMps1, EgMps1, CpMps1, and MtMps1). The structures of the DOC_CYCLIN_1, DOC_MAPK_1, and LIG_MAD2 motifs in AaMps1 were observed by tridimensional model analyses (Fig 3B).

### Effects of Mps1 Inhibition on Cellular Growth of Embryogenic Suspension Cultures

**Fig 4:**
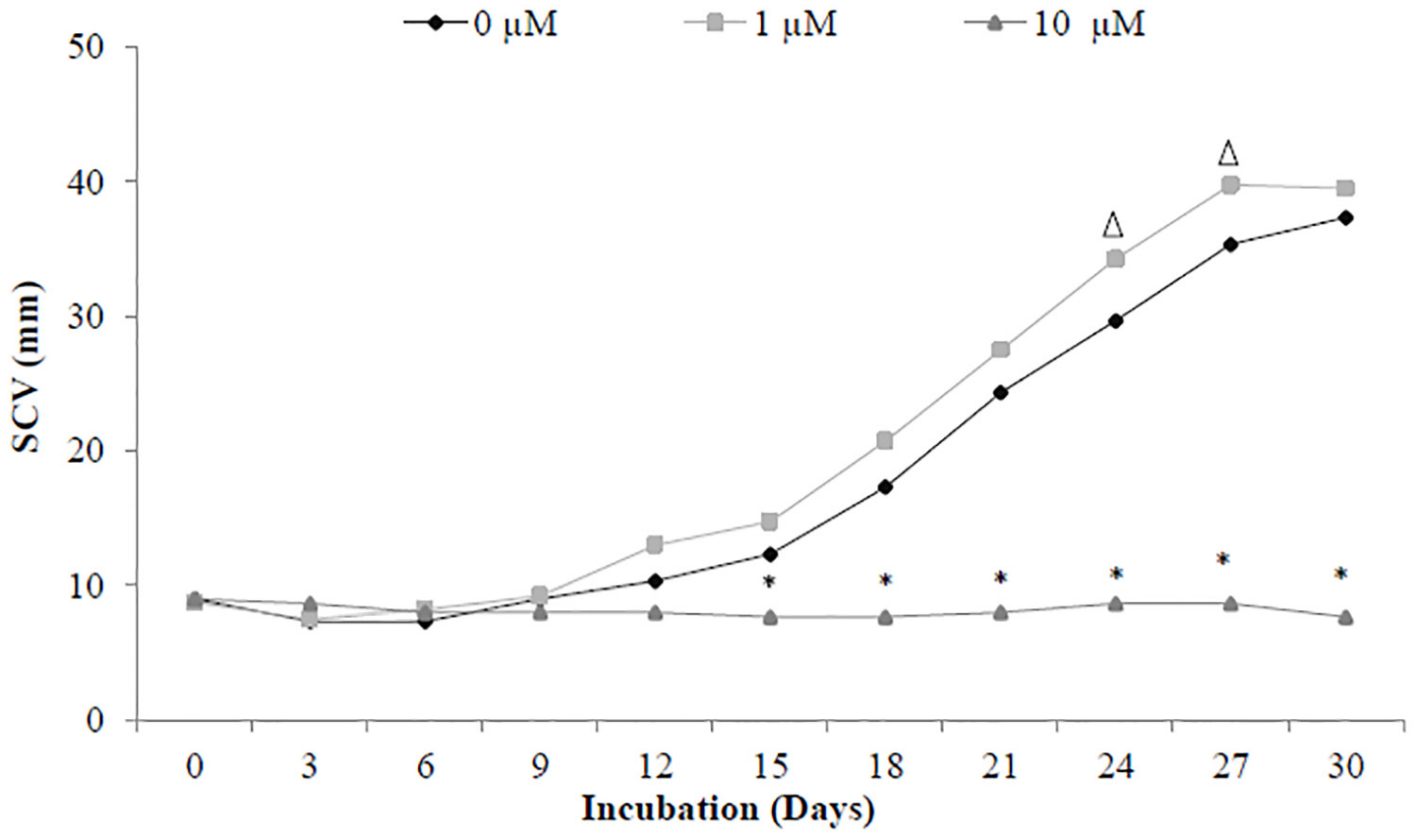
Cellular growth curve. Growth curve by settled cell volume (SCV) analyzes in embryogenic suspension cultures of *A*. *angustifolia* incubated with different concentrations (0, 1, and 10 μM) of Mps1 inhibitor SP600125, during 30 days of incubation. Triangles denote significant differences (*P* < 0.01) between control and 1 μM Mps1 inhibitor, and asterisks denote significant differences (*P* < 0.01) comparing 10 μM Mps1 inhibitor with the control and 1 μM Mps1 inhibitor treatments according to Tukey's test (n = 3; coefficient of variation = 14.5%).

Through SCV analysis (Fig 4), it was possible to observe the inhibition of cellular growth in *A*. *angustifolia* embryogenic suspension cultures treated with the Mps1 inhibitor at 10 μM, without significant differences in the incubation times. However, the cellular growth of embryogenic suspension cultures incubated in the control and 1 μM Mps1 inhibitor treatments increased during the incubation times, enabling the identification of the lagging (until the 12^th^ day), exponential (from the 15^th^ day), and stationary (27 days) phases (Fig 4).

**Fig 5:**
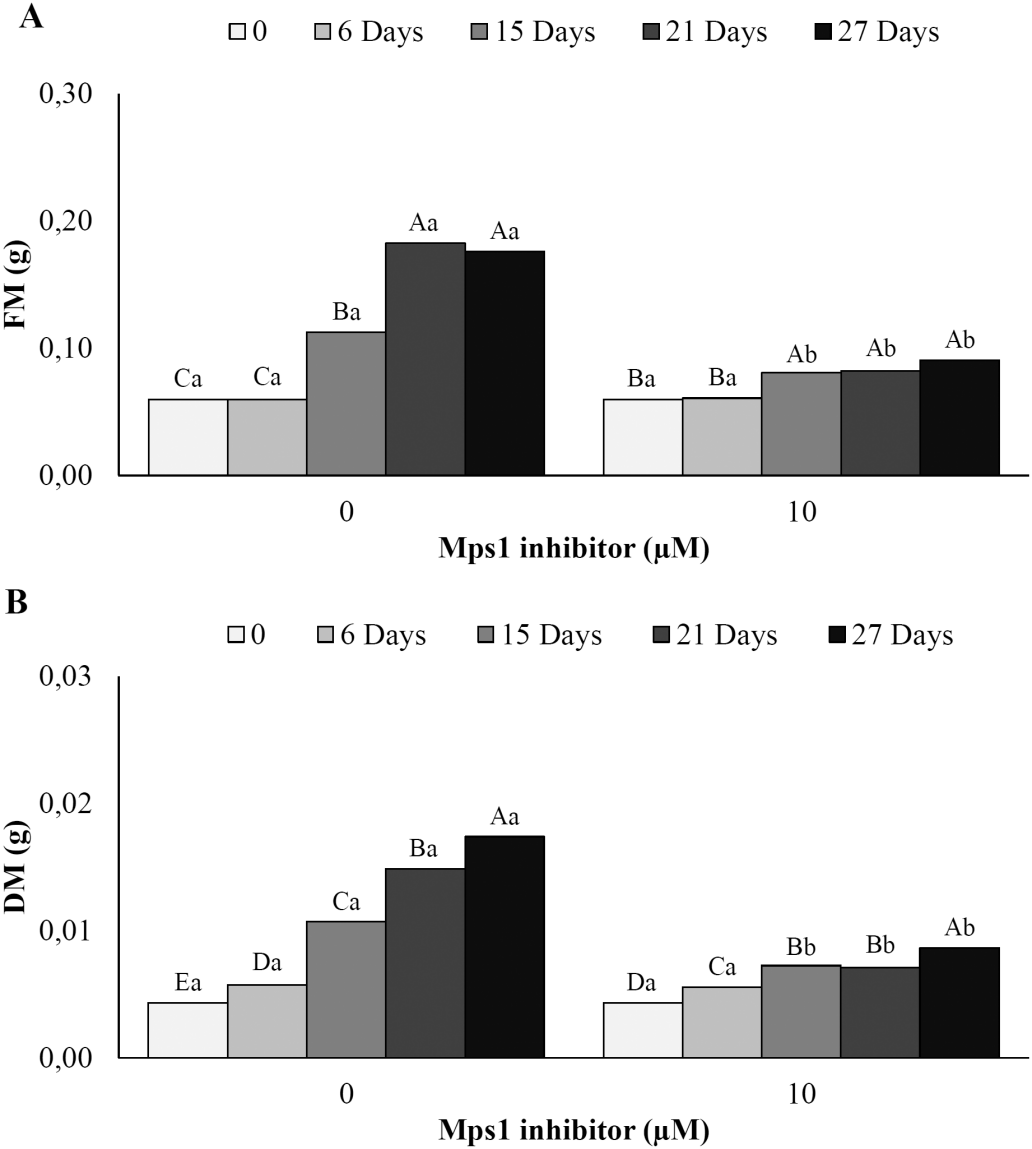
Mass increment (g) in *A*. *angustifolia* embryogenic suspension cultures. (A) FM and (B) DM values in embryogenic suspension cultures before (0) and after 6, 15, 21, and 27 days of incubation in MSG basic culture medium with (10 μM) or without Mps1 inhibitor SP600125. Lowercase letters denote significant differences (*P* < 0.01) between treatments for each day of incubation. Capital letters denote significant differences (*P* < 0.01) in the same treatment during incubation. Means followed by different letters are significantly different (*P* < 0.01) according to Tukey's test. CV = coefficient of variation (n = 6; CV FM = 10.3%; CV DM = 7.3%).

Cellular growth, in terms of the FM and DM increments in embryogenic suspension cultures during incubation, was affected by the Mps1 inhibitor. Beginning at 15 days of incubation, growth inhibition according to FM (Fig 5A) and DM (Fig 5B) analysis was observed in the presence of the Mps1 inhibitor. In addition, embryogenic suspension cultures showed a significant increase in the FM increment beginning on the 15^th^ day of incubation in the control treatment (Fig 5A). The DM increment in the control treatment was significant and progressive from the 6^th^ day until the end of incubation (Fig 5B).

### Effects of Mps1 Inhibition on PEM Morphology

**Fig 6:**
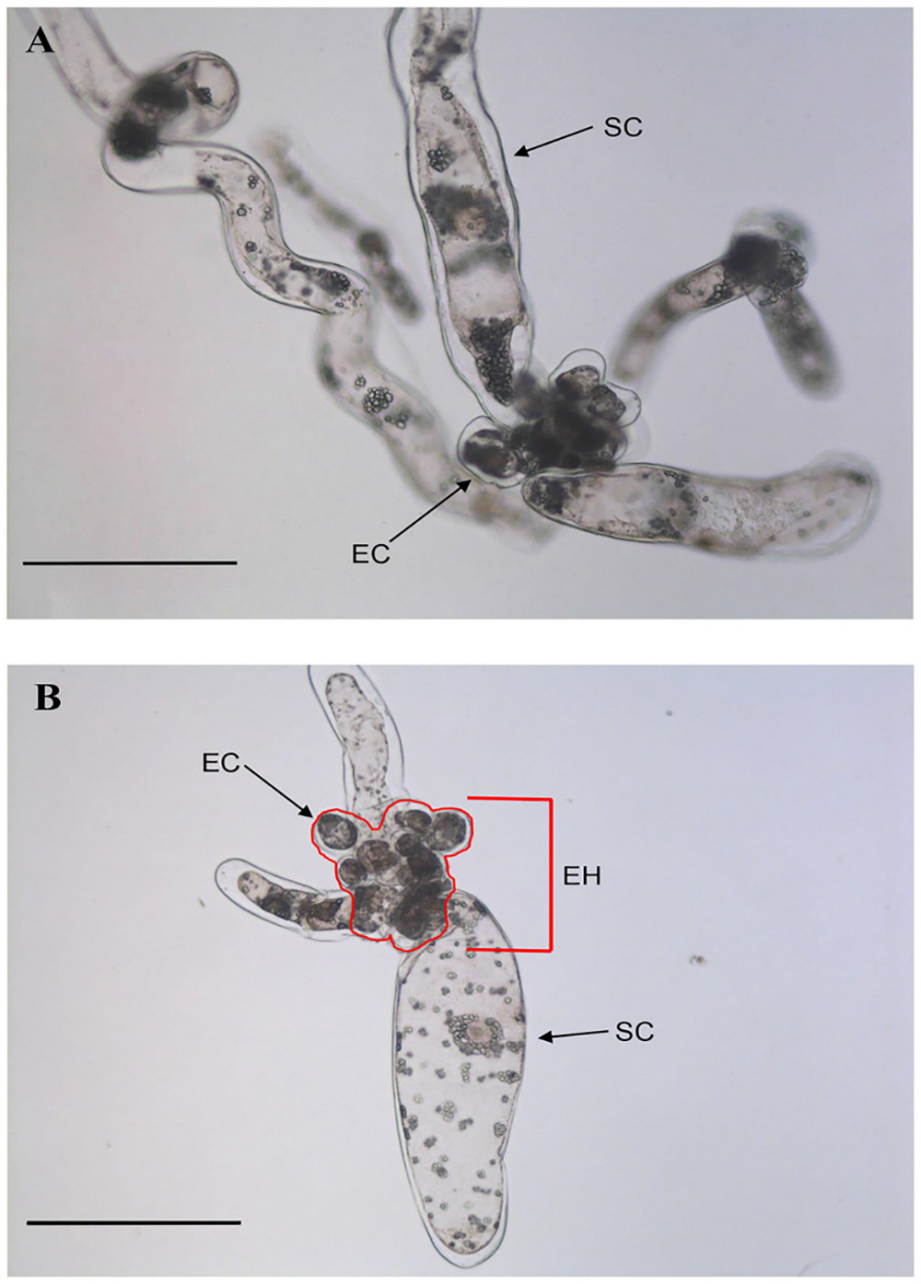
The morphology of *A*. *angustifolia* PEMs in cell suspension culture. Morphological features of PEMs after 15 days of incubation in MSG basic culture medium without (A) or with the Mps1 inhibitor SP600125 (10 μM) (B). EH = embryonal head; EC = embryogenic cells; SC = suspensor cells. Bars = 200 μm.

The morphology of PEMs was affected by the addition of 10 μM Mps1 inhibitor compared to the control treatment (Fig 6). These PEMs contain two types of cells: embryogenic cells (EC), which are grouped to form the embryonal head (HC), and the suspensor cells (SC). Embryogenic cells are isodiametric, with an evident nucleus, while suspensor cells are elliptic, being elongated and oblong (Fig 6).

**Fig 7:**
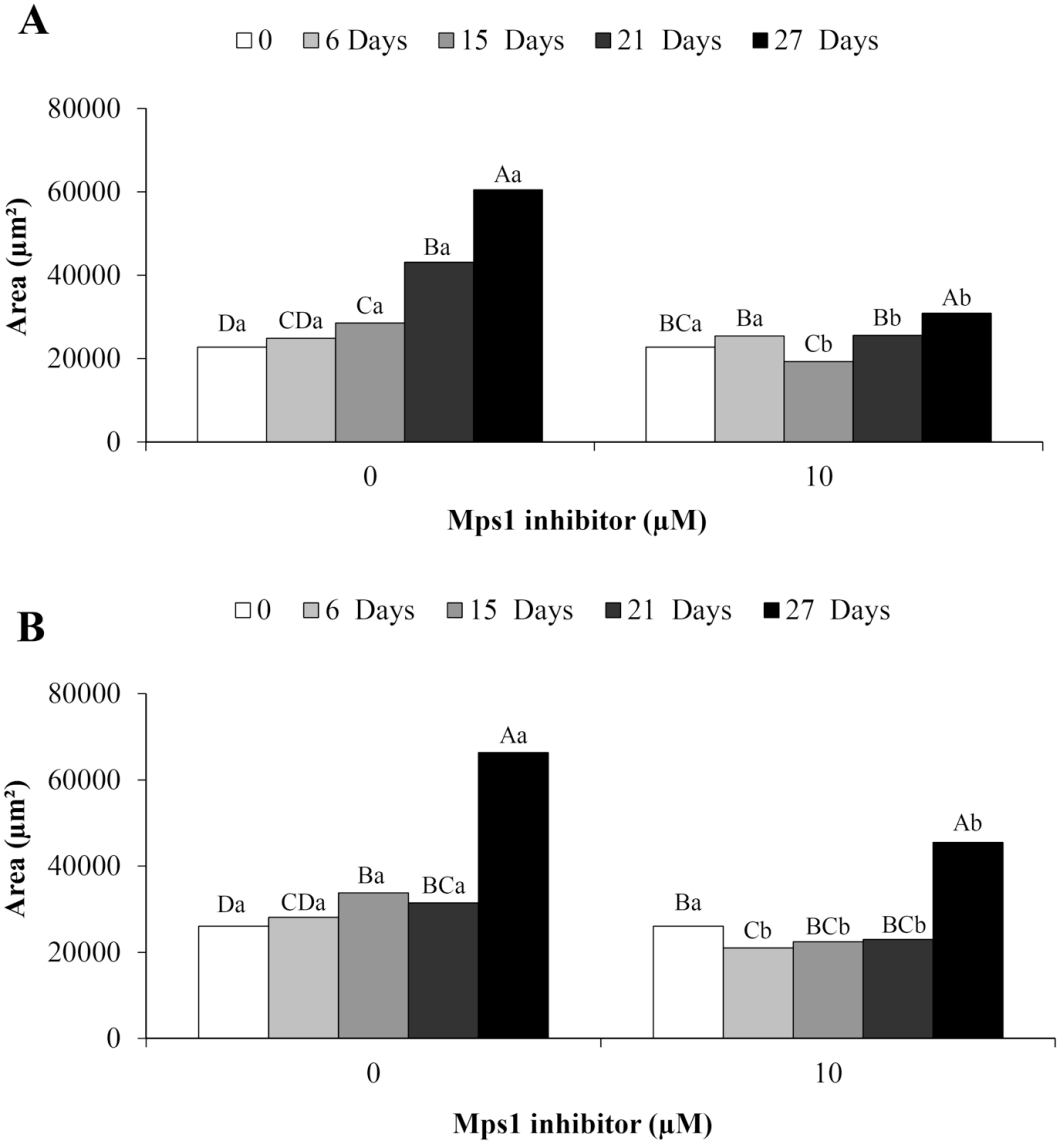
Analyses of PEM area. The group of embryogenic cells from the embryonal head of PEMs (A) and suspensor-type cells (B) from embryogenic suspension culture of *A*. *angustifolia* before (0) and after 6, 15, 21, and 27 days of incubation in MSG basic culture medium with (10 μM) or without the Mps1 inhibitor SP600125. Lowercase letters denote significant differences (P < 0.01) between treatments for each day of incubation. Capital letters denote significant differences (P < 0.01) in the same treatment during incubation. Means followed by different letters are significantly different (P < 0.01) according to Tukey's test. CV = coefficient of variation (n = 10; CV embryonal head = 13%; CV suspensor cells = 12%).

**Fig 8:**
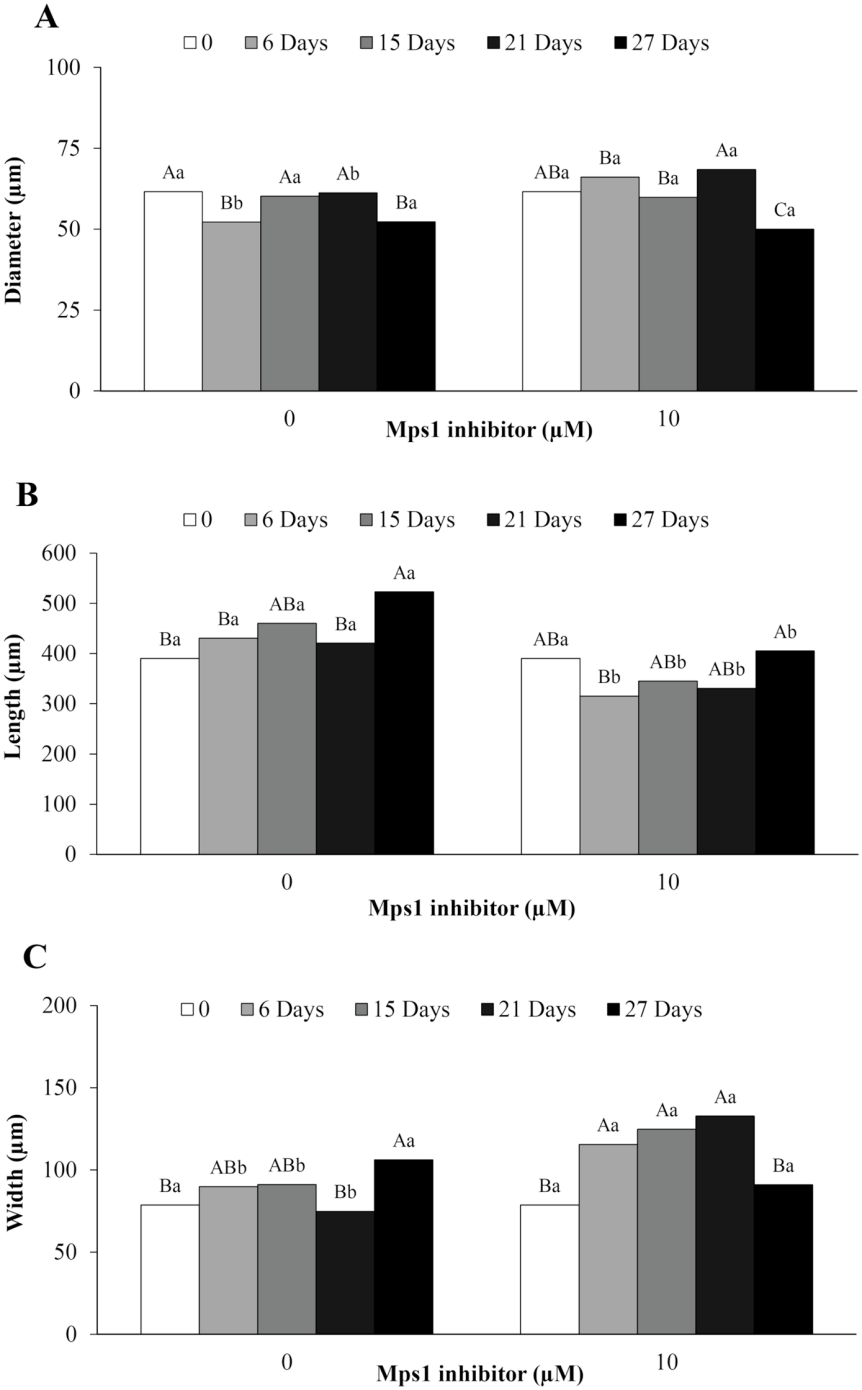
Analyses of cell size. Diameter of embryogenic cells (A) and length (B) and width (C) of suspensor cells from embryogenic suspension cultures of *A*. *angustifolia* before (0) and after 6, 15, 21, and 27 days of incubation in MSG basic culture medium with (10 μM) or without the Mps1 inhibitor SP600125. Lowercase letters denote significant differences (P < 0.01) between treatments for each day of incubation. Capital letters denote significant differences (P < 0.01) in the same treatment during incubation. Means followed by different letters are significantly different (P < 0.01) according to Tukey's test. CV = coefficient of variation (n = 50; CV diameter of embryogenic cells = 22.7%; CV length of suspensor cells = 35.2%; CV width of suspensor cells = 45.3%).

The embryogenic cells from the embryonal head of PEMs had significantly greater area from the 15^th^ to 27^th^ day in the control compared with those treated with 10 μM Mps1 inhibitor (Fig 7A), while the individual cells in the two treatments showed similar diameters during incubation (Fig 8A). On the other hand, the morphology of suspensor cells was affected by the addition of Mps1 inhibitor, showing changes in the area (Fig 7B) as well as the length (Fig 8B) and width (Fig 8C). Beginning on the 6^th^ day of incubation, the addition of Mps1 inhibitor reduced the area (Fig 7B) and length (Fig 8B) of suspensor cells in comparison to control cells. However, suspensor cells showed a significant increase in width from the 6^th^ to the 21^st^ day of incubation (Fig 8C).

### Identification and Quantification of the AaMps1 Protein

Mass spectrometry analyses compared the AaMps1 protein obtained by *in silico* analyses with the *Araucaria* transcriptome database [22,23], resulting in 81.66% sequence coverage. These results confirm the presence of the Mps1 protein in *A*. *angustifolia* embryogenic suspension cultures (Table 2). Furthermore, the AaMps1 protein was highly similar to the AtMps1 protein (gi | 28416703), with 78.76% sequence coverage, indicating a strong homology between the AaMps1 and AtMps1 proteins (Table 2).

**Table 2:** AaMps1 protein identification.

Parameters	AaMps1	AtMps1
**Score**	191.25	226.92
**Coverage (%)**	81.6602	78.6358
**mW (Da)**	113944	86323
**pI (pH)**	6.7	6.44

AaMps1 protein identification by HDMS^E^ (data-independent acquisition, with ion mobility) mass spectrometry in embryogenic suspension cultures of *A*. *angustifolia* incubated without Mps1 inhibitor SP600125, compared with *in silico* predicted protein sequence of AaMps1 (from *A*. *angustifolia* transcriptome database) and AtMps1 (*A*. *thaliana*) protein.

**Fig 9:**
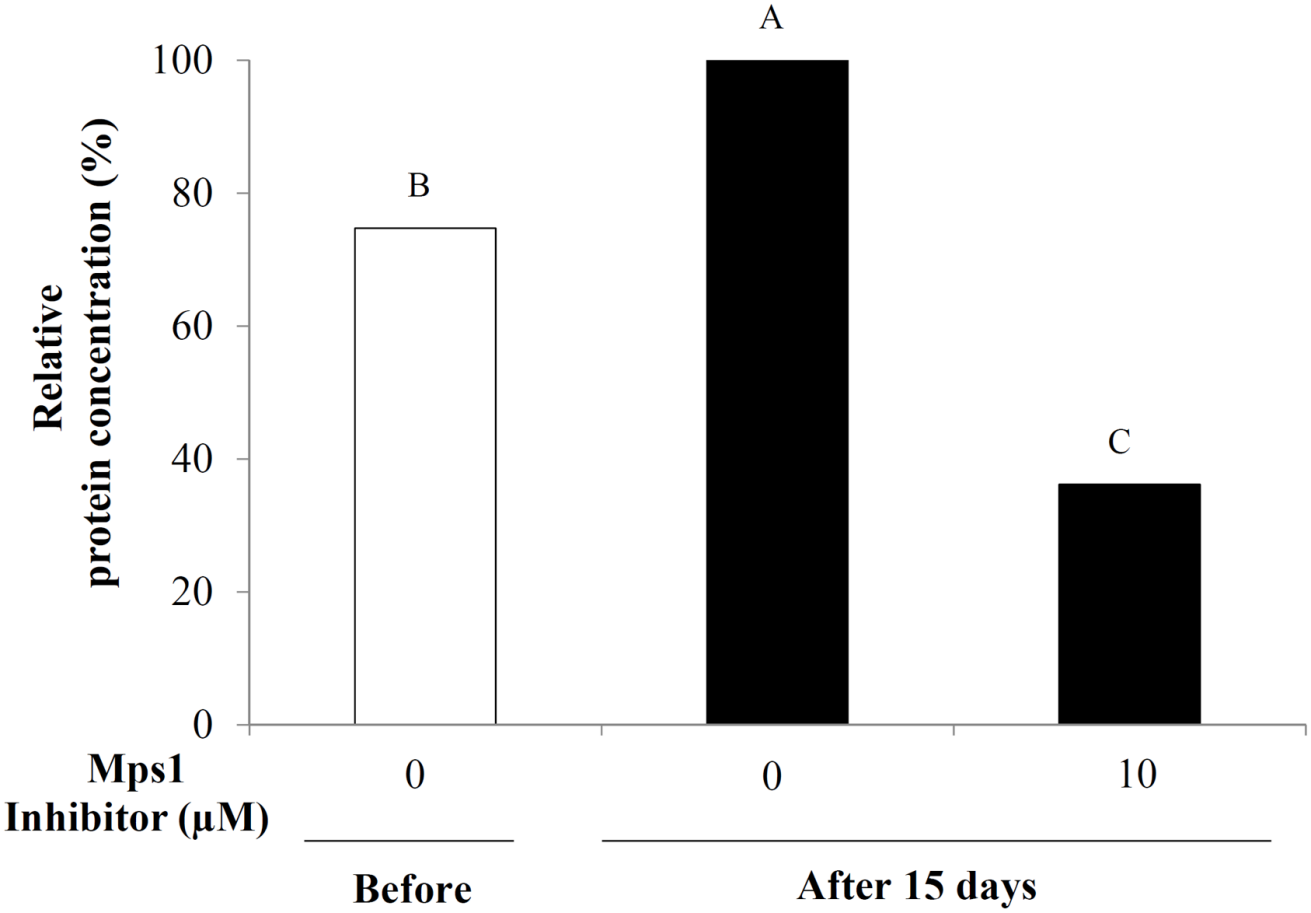
Quantification of the AaMps1 protein. Relative concentration (%) of AaMps1 protein by HDMS^E^ (data-independent acquisition, with ion mobility) mass spectrometry analysis in embryogenic suspension cultures of *A*. *angustifolia* before (0) and after 15 days of incubation in MSG basic culture medium with (10 μM) or without Mps1 inhibitor SP600125. Means followed by different letters are significantly different (*P* < 0.01) according to Tukey's test. (n = 3; Coefficient of variation = 14.1%).

In addition, embryogenic suspension cultures at 15 days of incubation without Mps1 inhibitor (control) demonstrated a significant increase in the amount of AaMps1 compared with those analyzed before incubation (time 0). Furthermore, treatment with the Mps1 inhibitor (10 μM) induced a decrease in the amount of AaMps1 protein at 15 days of incubation compared with the control at 15 days of incubation and with embryogenic cultures before incubation (Fig 9).

## Discussion

Our results show the presence of the Mps1 protein in the gymnosperm species *A*. *angustifolia*, designated AaMps1. AaMps1 is homologous with the Mps1 proteins of other species, including the kinase domain that is highly conserved in eukaryotes. Our work confirmed the existence of the AaMps1 protein in embryogenic cultures by mass spectrometry analysis, demonstrating high coverage of the *in silico* predicted protein sequence of AaMps1 and with the AtMps1 (*A*. *thaliana*) protein.

Among the analyzed species, *A*. *trichopoda* (AbMps1) presented more similarities to AaMps1 in terms of residue number (Fig 1 and S1 Fig). This result may be related to the origin of *A*. *trichopoda*; this species is a member of an ancient lineage and is a unique and valuable reference that facilitates the interpretation of major genomic events in the evolution of flowering plants [41]. In addition, the AaMps1 protein shows similarities with AtMps1, the recently described Mps1 protein in plants [18] as well as other plants, such as *E*. *grandis*, *C*. *papaya*, and *M*. *truncatula* (S1 Fig). However, the numbers of residues from the Mps1 protein of these species are lower compared with that of *A*. *angustifolia* (AaMps1) and *A*. *trichopoda* (AbMps1). This result could explain the larger size of the AaMps1 protein in relation to AtMps1, given that AaMps1 presented more similarities with AbMps1 from *A*. *trichopoda* in comparison with *A*. *thaliana*. *A*. *trichopoda* is an ancestral angiosperm species for which the genome has been published, making this species a pivotal reference for understanding genome and gene family evolution throughout angiosperm history [41].

Furthermore, AaMps1 has several structural features present in the Mps1 of all analyzed species, such as phosphorylation sites, DFG motifs, and the threonine triad (Figs 2 and 3; Table 1). Events such as the phosphorylation and autophosphorylation of Mps1 by other proteins and Mps1-mediated phosphorylation are crucial for the correct location and activity of Mps1 in cell cycle control [42–44]. Autophosphorylation on three fundamental threonine residues (the threonine triad) in the Mps1 loop is necessary to activate this protein in humans (hMps1). Studies related to phosphorylation site mapping and mutation analysis in hMps1 indicate that three residues—T675, T676, and T686—may be modified by autophosphorylation, given that the phosphorylation of T676 within the hMps1 activation loop is important for full kinase activity [43]. These three important residues in hMps1 are present in *A*. *thaliana* as T579, T580 and T590 [18], and they were shown to be conserved in *A*. *angustifolia* as T870, T871 and T881.

Other characteristic features of Mps1 were also observed in AaMps1 in terms of interaction regions with other proteins (Fig 3). Some motifs observed in AaMps1, such as DOC_CYCLIN_1, DOC_MAPK_1, LIG_MAD2, TRG_NES_CRM1_1, TRG_NLS_MonoExtC, DOC_PP1_RVXF_1, and MOD_PIKK_1 (Fig 3A), could potentially mediate interactions with cyclins, MAD2, the anaphase-promoting complex/cyclosome (APC/C), and MAPK-cell cycle regulators [44,45]. These motifs were similar to those of the other analyzed species, and some of the motifs have also been reported in Mps1 proteins in other plants, such as *A*. *thaliana* (AtMps1), *Populus trichocarpa* (PtMps1), *Ricinus communis* (RcMps1), *Oryza sativa* (OsMps1), *Sorghum bicolor* (SbMps1), and *Zea mays* (ZmMps1) [18]. These regions interact through short amino acid modules (linear motifs), which are frequently identified as regulatory protein parts that provide interactions and bind with other proteins, modifying their structures and activities [29].

In addition, some interactions between Mps1 and other proteins that were observed through the predicted motifs have been shown in previous studies related to Mps1, such as the presence of the LIG_MAD2 motif, thus suggesting an interaction between Mps1 and MAD2 proteins in *A*. *angustifolia* (Fig 3). Experiments using human HeLa cells verified that Mps1 kinase promotes C-MAD2 production and subsequently leads the mitotic checkpoint complex (MCC) to activate the SAC; additionally, impaired inhibition of the Mps1, BubR1-MAD2 interaction has been shown, as well as the incorporation of MCC into MAD2 [44]. During the cell cycle, the increased phosphorylation of Mps1 at M phase is dependent on MAPK. MAPK is required for the SAC, and the phosphorylation of cell division control protein 20 (Cdc20) by MAPK is required for Cdc20 to associate with spindle-checkpoint proteins [45]. Herein, we identify the DOC_MAPK_1 motif in AaMps1, which may be another target for MAPK in the spindle checkpoint [45]. Therefore, the sequence of the AaMps1 protein shows some motifs and phosphorylation sites with higher similarities to those of other species, confirming the identity of this protein in *A*. *angustifolia*.

Our results showed that the inhibition of AaMps1 affects the cellular growth (Figs 4 and 5) and PEM morphology of embryogenic suspension cultures in *A*. *angustifolia* (Figs 6, 7 and 8). These results suggest that the Mps1 protein is present in this species and that the inhibition of this protein with the Mps1 inhibitor can arrest the cell cycle. In addition, a decrease in the amount of AaMps1 protein, which was induced by the inhibitor, showed a strong correlation with the cellular growth reduction observed in *A*. *angustifolia* embryogenic suspension cultures (Fig 9). SP600125 competes for the ATP binding site on Mps1 and thus prevents the activity of this protein kinase during cell cycle control in plants [18]. The inhibition of AaMps1 in *A*. *angustifolia* embryogenic suspension cultures reduces cellular growth, and it may be useful for understanding cell cycle control in gymnosperm somatic embryogenesis as well as for further studies on improving somatic embryo development.

## Conclusions

This work has demonstrated the identification of Mps1 protein in *A*. *angustifolia* (AaMps1), showing that inhibition by the Mps1 inhibitor SP600125 affects the development of embryogenic cultures, reducing cellular growth, PEM morphology, and the amount of AaMps1 protein. Mass spectrometry analysis showed high homology with the AaMps1 predicted protein, obtained by *in silico* analyses with the *Araucaria* transcriptome database, and with the AtMps1 protein.

## Supporting Information

S1 Fig Multiple sequence alignment. Kinase domain in Mps1 proteins from *A*. *angustifolia* (AaMps1), *A*. *thaliana* (AtMps1), *A*. *thicopoda* (AbMps1), *E*. *grandis* (EgMps1), *C*. *papaya* (CpMps1), and *M*. *truncatula* (MtMps1). The black arrows indicate amino acids important for interaction with the inhibitor that are also conserved between hMps1, AaMps1 and other plant species. The blue arrow indicates the conserved DFG motifs. The red arrow indicates the conserved threonine residues.
(TIF)

S2 Fig Tridimensional modeling and overlap of AaMps1 kinase domains and AtMps1. AaMps1 (cyan) in *A*. *angustifolia* and AtMps1 (green) in *A*. *thaliana*.
(TIF)

S3 Fig Tridimensional modeling and overlap of AaMps1 kinase domains and hMps1. AaMps1 (cyan) in *A*. *angustifolia* and hMps1 (pink) in humans.
(TIF)

S1 Table Sequence information. (DOCX)
