# Supplementary figures and images for: Mps1 (Monopolar Spindle 1) Protein Inhibition Affects Cellular Growth and Pro-Embryogenic Masses Morphology in Embryogenic Cultures of Araucaria angustifolia (Araucariaceae)
Source: PLoS One. 2016 Apr 11;11(4):e0153528. doi: 10.1371/journal.pone.0153528 (PMC4827878; doi:10.1371/journal.pone.0153528)

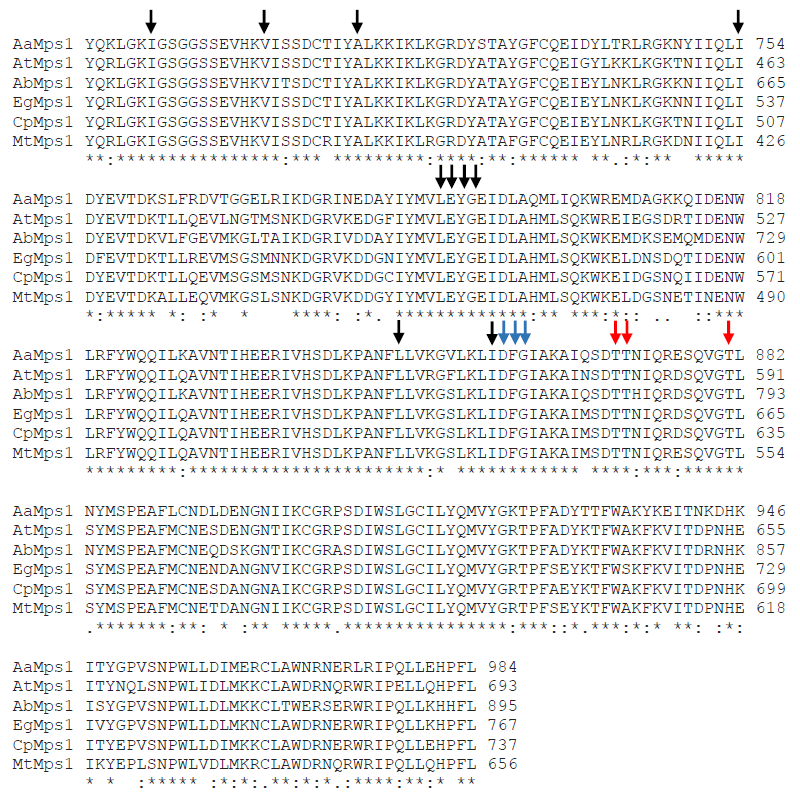

Supplement: S1 Fig — Kinase domain in Mps1 proteins from A. angustifolia (AaMps1), A. thaliana (AtMps1), A. thicopoda (AbMps1), E. grandis (EgMps1), C. papaya (CpMps1), and M. truncatula (MtMps1). The black arrows indicate amino acids important for interaction with the inhibitor that are also conserved between hMps1, AaMps1 and other plant species. The blue arrow indicates the conserved DFG motifs. The red arrow indicates the conserved threonine residues. (TIF) [file pone.0153528.s001.tif]

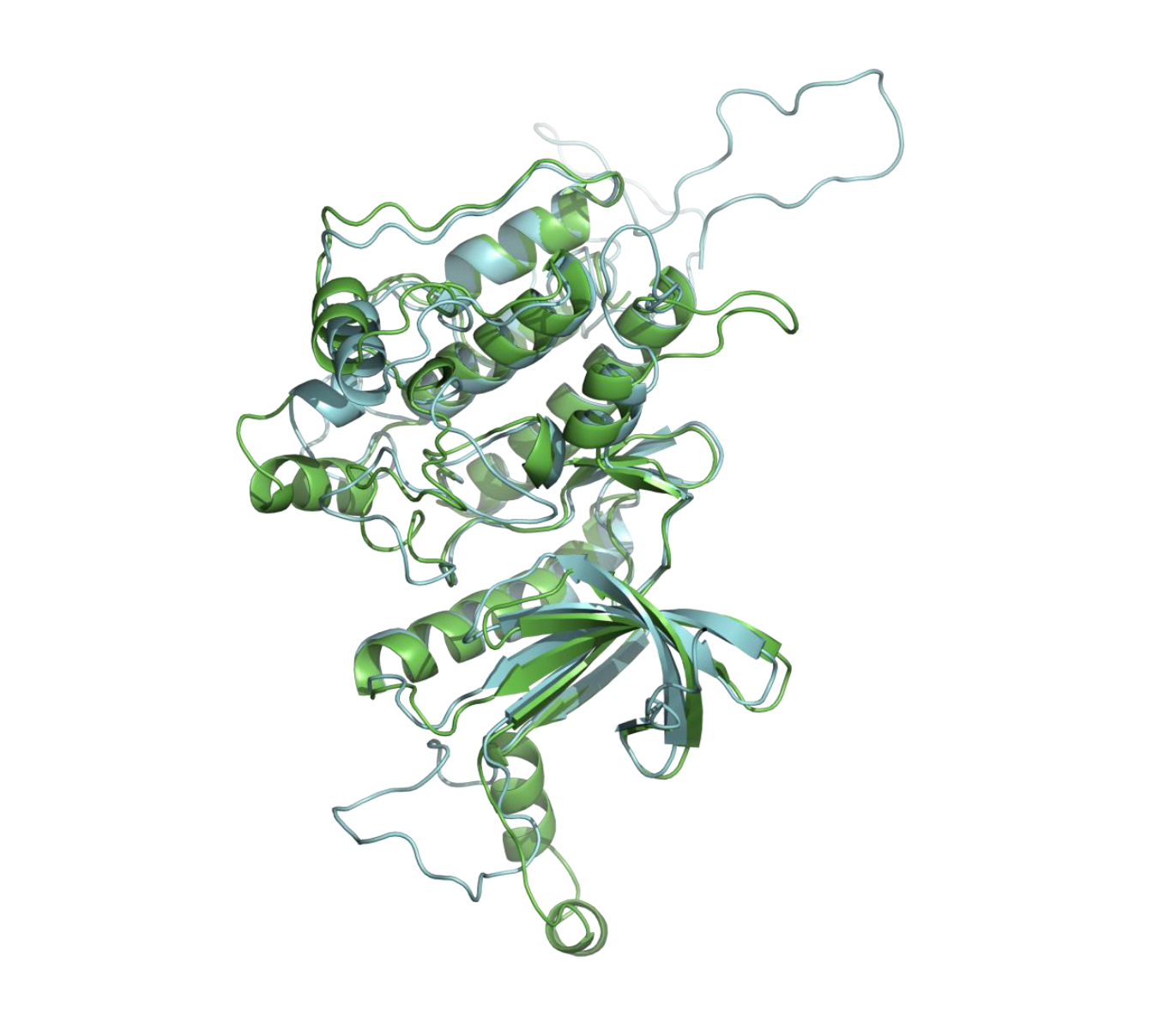

Supplement: S2 Fig — AaMps1 (cyan) in A. angustifolia and AtMps1 (green) in A. thaliana. (TIF) [file pone.0153528.s002.tif]

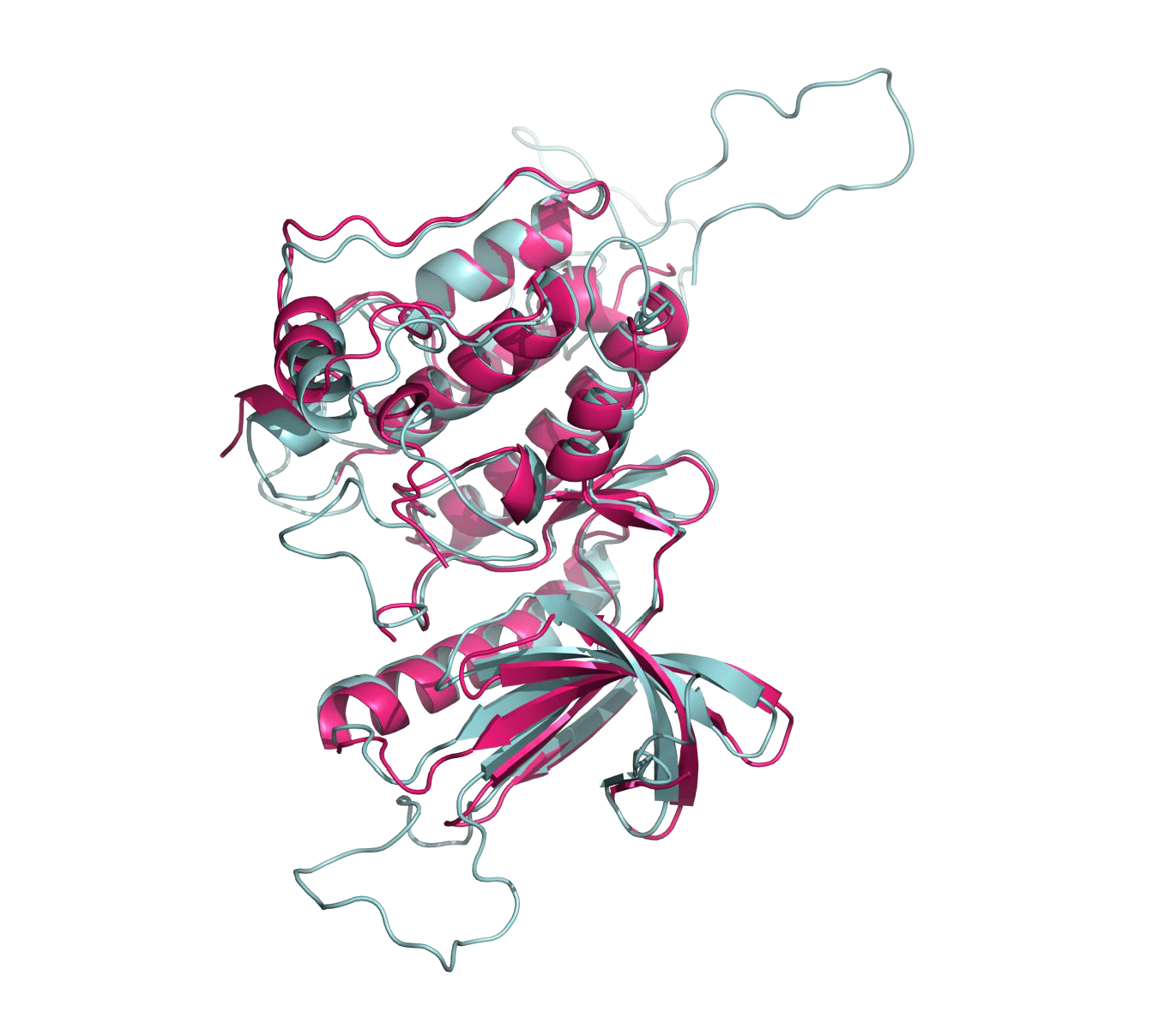

Supplement: S3 Fig — AaMps1 (cyan) in A. angustifolia and hMps1 (pink) in humans. (TIF) [file pone.0153528.s003.tif]
