# Supplementary material for: Mps1 (Monopolar Spindle 1) Protein Inhibition Affects Cellular Growth and Pro-Embryogenic Masses Morphology in Embryogenic Cultures of Araucaria angustifolia (Araucariaceae)
Source: PLoS One. 2016 Apr 11;11(4):e0153528. doi: 10.1371/journal.pone.0153528 (PMC4827878; doi:10.1371/journal.pone.0153528)
Supplement: S1 Table — (DOCX) [file pone.0153528.s004.docx]

**S1 Table.** Sequence information.

| **Species** | **Name** | **Accession number** | **Database** |
| --- | --- | --- | --- |
| *Amborella trichopoda* | AbMps1-like | 586646077 | NCBI |
| *Aquilegia coerulea* | AcMps1-like | 22026026 | Phytozome |
| *Arabidopsis lyrata* | AlMps1-like | 55089163 | Phytozome |
| *Arabidopsis thaliana* | AtMps1 | 19651099 | Phytozome |
| *Arabidopsis thaliana* | MPKKK3 | AT1G53570.1 | TAIR |
| *Arabidopsis thaliana* | MAPKKK19 | AT5G67080.1 | TAIR |
| *Araucaria angustifolia* | AaMps1 | comp44392_c0_seq1 | Araucaria |
| *Boechera stricta* | BsMps1-like | 30674076 | Phytozome |
| *Brachypodium distachyon* | BdMps1-like | 31132433 | Phytozome |
| *Brassica rapa* | BrMps1_like2 | 30632638 | Phytozome |
| *Brassica rapa* | BrMps1-like | 30635976 | Phytozome |
| *Capsella grandiflora* | CgMps1-like | 28896987 | Phytozome |
| *Capsella rubella* | CrMps1-like | 20905877 | Phytozome |
| *Carica papaya* | CpMps1-like | 16418293 | Phytozome |
| *Citrus clementina* | CcMps1-like | 20785857 | Phytozome |
| *Citrus clementina* | CcMps1-like2 | 20785858 | Phytozome |
| *Cucumis sativus* | CsMps1-like | 16971492 | Phytozome |
| *Cucumis sativus* | CsMps1-like2 | 16971493 | Phytozome |
| *Eucalyptus grandis* | EgMps1-like | 23584928 | Phytozome |
| *Eutrema salsugineum* | EsMps1-like | 20192414 | Phytozome |
| *Fragaria vesca* | FvMps1-like | 27261715 | Phytozome |
| *Glycine max* | GmMps1-like | 30487385 | Phytozome |
| *Glycine max* | GmMps1-like2 | 30553833 | Phytozome |
| *Glycine max* | GmMps1-like3 | 30487385 | Phytozome |
| *Glycine max* | GmMps1-like4 | 30504360 | Phytozome |
| *Gossypium raimondii* | GmMps1-like5 | 30533762 | Phytozome |
| *Gossypium raimondii* | GrMps1-like | 26765252 | Phytozome |
| *Gossypium raimondii* | GrMps1-like2 | 26760976 | Phytozome |
| *Linum usitatissimum* | LusMps1-like | 23181249 | Phytozome |
| *Malus domestica* | MdMps1-like | 22634014 | Phytozome |
| *Manihot esculenta* | MeMps1-like | 17993060 | Phytozome |
| *Medicago trunculata* | MtMps1-like | 31054629 | Phytozome |
| *Medicago trunculata* | MtMps1-like2 | 31091020 | Phytozome |
| *Mimulus guttatus* | MgMps1-like | 28944327 | Phytozome |
| *Mimulus guttatus* | MgMps1-like2 | 28926427 | Phytozome |
| *Oryza sativa* | OsMps1-like | 24124978 | Phytozome |
| *Panicum virgatum* | PvMps1-like | 30239330 | Phytozome |
| *Phaseolus vulgaris* | PhvMps1-like | 27146011 | Phytozome |
| *Phaseolus vulgaris* | PhvMps1-like2 | 27146012 | Phytozome |
| *Populus trichocarpa* | PtrMps1-like | 27024355 | Phytozome |
| *Populus trichocarpa* | PtrMps1-like2 | 27029976 | Phytozome |
| *Prunus persica* | PpMps1-like | 17662821 | Phytozome |
| *Ricinus communis* | RcMps1-like | 16813011 | Phytozome |
| *Salix purpurea* | SpMps1-like | 31403193 | Phytozome |
| *Salix purpurea* | SpMps1-like2 | 31428444 | Phytozome |
| *Salix purpurea* | SpMps1-like3 | 31407206 | Phytozome |
| *Solanum tuberosum* | StMps1-like | 24407254 | Phytozome |
| *Sorghum bicolor* | SbMps1-like | 28395238 | Phytozome |
| *Theobroma cacao* | TcMps1-like | 27459214 | Phytozome |
| *Vitis vinifera* | VvMps1-like | 17821444 | Phytozome |
| *Zea mays* | ZmMps1-like | 31017286 | Phytozome |
